# Identification of Compounds That Inhibit IGF-I Signaling in Hyperglycemia

**DOI:** 10.1155/2009/267107

**Published:** 2010-01-06

**Authors:** Laura A. Maile, Lee B. Allen, Umadevi Veluvolu, Byron E. Capps, Walker H. Busby, Michael Rowland, David R. Clemmons

**Affiliations:** Division of Endocrinology, The University of North Carolina at Chapel Hill, NC 27599-7170, USA

## Abstract

Increased responsiveness of vascular cells to the growth factor IGF-I has been implicated in complications associated with diabetes. Here we describe the development of an assay and screening of a library of compounds for their ability to accelerate cleavage of the transmembrane protein integrin-associated protein (IAP) thereby disrupting the association between IAP and SHPS-1 which we have shown as critical for the enhanced response of vascular cells to IGF-I. The cell-based ELISA utilizes an antibody that specifically detects cleaved, but not intact, IAP. Of the 1040 compounds tested, 14 were considered active by virtue of their ability to stimulate an increase in antibody-binding indicative of IAP cleavage. In experiments with smooth muscle and retinal endothelial cell cultures in hyperglycemic conditions, each active compound was shown to accelerate the cleavage of IAP, and this was associated with a decrease in IAP association with SHPS-1 as determined by coimmunoprecipitation of the proteins from cell lysates. As a consequence of the acceleration in IAP cleavage, the compounds were shown to inhibit IGF-I-stimulated phosphorylation of key signaling molecules including Shc and ERK1/2, and this in turn was associated with a decrease in IGF-I-stimulated cell proliferation. Identification of these compounds that utilize this mechanism has the potential to yield novel therapeutic approaches for the prevention and treatment of vascular complications associated with diabetes.

## 1. Introduction

Increased cellular responsiveness to insulin-like growth factor-I (IGF-I) has been implicated in several complications associated with diabetes including vascular complications such as atherosclerosis [1–3] and diabetic retinopathy [4–6] as well as other complications such as neuropathy [7–16]. Directly targeting IGF-I or its receptor; however, is likely to be associated with unwanted side effects. Our long-term goal is to develop therapeutic strategies that could specifically antagonize the effects of IGF-I associated with hyperglycemia yet preserve IGF-I's beneficial effects.

People with both type 1 and type 2 diabetes develop atherosclerosis at a significantly accelerated rate compared to non diabetics [17–19]. Recent studies have suggested that hyperglycemia plays a significant role in the acceleration of lesion initiation and progression in patients with both type 1 and type 2 diabetes [20–22]. Proliferative diabetic retinopathy (PDR) is characterized by the growth of unwanted blood vessels and intravitreous neovascularization (IVNV) [23]. Formation of these new blood vessels requires retinal endothelial cell (REC) proliferation and migration [24]. Hyperglycemia appears to contribute directly to both SMC proliferation and migration associated with atherosclerosis [25].and the neovascularization associated with PDR [21]. Insulin-like growth factor-I (IGF-I) stimulates SMC migration and proliferation and has therefore been implicated in the lesion progression [1–3]. Similarly various studies have implicated IGF-I as a contributor to the retinal neovascularization associated with PDR [4–6]. Both REC and SMC grown in high glucose are more responsive to the stimulatory effects of IGF-I compared to cells grown in normal glucose [26].

Activation of the intrinsic kinase activity of the IGF-I receptor (IGF-IR) is required to trigger downstream signaling events that lead to cellular proliferation. There is no difference in abundance or extent of IGF-IR activation between SMC grown in normal or high glucose; therefore, this does not account for the difference in response to IGF-I [26]. The proliferative response of both REC and SMC to IGF-I in hyperglycemia is dependent upon the interaction between the extracellular domains of two transmembrane proteins, integrin-associated protein (IAP) and SHP substrate 1 (SHPS-1) [27]. When IAP is bound to SHPS-1, tyrosine residues within the cytoplasmic domain of SHPS-1 are phosphorylated in response to activation of the IGF-IR [27]. Phosphorylation of these tyrosine residues is required for the recruitment of signaling molecules to SHPS-1 and their recruitment is required to elicit an increase in SMC proliferation in response to IGF-I [28, 29]. Our studies have shown that when REC and SMC are grown in 5 mM glucose conditions the extracellular domain of IAP is cleaved and the residual IAP fragment that remains cell-surface associated cannot bind SHPS-1 [30, 31]. When vascular cells are grown in high glucose (e.g., >12 mM), IAP is protected from cleavage and there is a significant increase in its interaction with SHPS-1. This in turn is associated with increased SHPS-1 phosphorylation, enhanced activation of downstream signaling events and increased cell proliferation in response to IGF-I [31, 32]. These observations led us to hypothesize that stimulation of IAP cleavage in the presence of hyperglycemia could be a novel strategy for the inhibition of IGF-I-stimulated REC and SMC proliferation.

The Juvenile Diabetes Research Foundation (JDRF) in conjunction with National Institutes of Health-National Institutes of Diabetes and Digestive Kidney Disease (NIDDK) funded a program to screen a panel of 1040 selected compounds for the prevention of cellular dysfunction in various cell types that are relevant to diabetic complications (a document containing a summary of the various assays that were developed and the outcomes is available online (http://www.t1diabetes.nih.gov/Investigator/Drug-ScreeningSummary-Final.doc)). In response to this program we designed, implemented and validated a cell-based ELISA that measured the ability of each compound to accelerate IAP cleavage in SMC grown in high glucose. In this study we describe the initial testing of the compounds leading to the identification of 14 compounds that were analyzed for their ability to inhibit IGF-I signaling in vascular cells.

## 2. Materials and Methods

### 2.1. Materials

Human, endotoxin-free, IGF-I was a gift from Genentech (South San Francisco, CA). Polyvinyl difluoride membrane (Immobilon P) was purchased from Millipore Corporation (Billerica, MA). Cl-Xposure autoradiographic film was obtained from Pierce (Rockford, IL). Fetal Bovine Serum, Dulbecco's modified medium, penicillin and streptomycin were purchased from Life Technologies, (Grand Island, NY). The monoclonal antiphosphotyrosine antibody (PY99) was purchased from Santa Cruz (Santa Cruz, CA). The anti-Shc and phospho/total ERK1/2 antibodies were purchased from BD Transduction Laboratories (Lexington, KY). The anti-SHPS-1 antibody was purchased from Upstate Cell Signaling Solutions (Charlottesville, VA). All other reagents were purchased from Sigma Chemical Company (St Louis, MO) unless stated.

### 2.2. Anti-IAP Antibodies

The anti-IAP monoclonal antibody, B6H12, was purified from a specific cell line derived from a B-cell hybridoma as we have described previously [33]. The anti-IAP antibodies were generated by conjugating a peptide to keyhole limpet hemagglutinin and used for immunization as we have described previously [34]. Antibody R569 was generated using a peptide homologous to amino acids 41–61 of the extracellular domain of IAP (KGRDIYTFDGALNKSTVPTC) [31]; R593 was generated using a peptide homologous to amino acids 74–96 of the extracellular domain and R250 was generated using a peptide homologous to amino acids 279–292 [31]. Serum from a nonimmunized rabbit was used as control antiserum.

### 2.3. Porcine SMC

SMC were isolated from porcine aortic explants by a modification of the protocol by Ross [35] as we have described previously [26]. Following isolation SMC were maintained in either high glucose growth medium (HG-GM) (DMEM containing 4500 g/L (25 mM) glucose) or normal glucose (NG-GM) (DMEM containing 900 g/L of glucose (5 mM)) plus 10% fetal bovine serum (FBS), penicillin (1000 U/mL), streptomycin (160 *μ*g/mL) and 0.5 mM sodium pyruvate. SMC were fed every 3 days with either HG- or NG-GM and were passed every 7 days in the appropriate medium. All experiments were performed on SMC between passage number 4 and 10. We have previously determined that SMC cultured under these two different conditions do not differ significantly in their differentiation status [26]. To control for differences in osmolarity, mannitol (19.5 mM) was added to the NG-serum-free medium [26]. We have determined previously that the presence or absence of mannitol does not influence the responses of SMC grown in NG [26].

### 2.4. Retinal Endothelial Cells

Primary bovine RECs were obtained from VEC Technologies Inc (Rensselaer, NY). Stock cultures of RECs were grown in a defined EC growth medium (MCDB-131 complete) supplied by VEC Technologies Inc which contains 5 mM glucose. Stock cultures of RECs were grown in 10 cm dishes precoated with 50 *μ*g/mL fibronectin in PBS for 30 minutes at 37°C. To examine the effects of the compounds in high glucose the medium was supplemented to 25 mM glucose as we have described previously [30].

### 2.5. Primary Screening of Compounds: Assay Design and Validation

SMC were maintained in 10 cm dishes in HG-GM. For individual experiments SMC were plated in 96 well plates (Falcon) in HG-GM at a density of 5000 cells per well. SMCs were grown to confluency over a period of 5 days with no media change.

#### 2.5.1. Antibodies

Positive control: The monoclonal antibody B6H12 binds equally well to IAP in both normal and high glucose, that is, it does not distinguish between intact or cleaved IAP. Therefore the binding of this antibody was used to control for total amount of IAP [31]. Negative Control: Antibody R250 is a polyclonal antibody raised in rabbits with a peptide homologous to amino acids 279 to 292 of human IAP (VASDHKTIQPPRNN). This region of IAP is the most C-terminal region of the cytoplasmic domain and therefore served as a negative control since this region of IAP is not surface exposed and can not bind to IAP when cells are intact. Test Antibody: Antibody R593 is a polyclonal antibody raised in rabbits against a peptide homologous to amino acids 74 to 96 of the mature human IAP sequence (KGDASLKMDKSDAVSHTGNYT). R593 binds to cleaved IAP with a higher affinity than to that of intact IAP due to exposure of a neo-epitope following cleavage.

Characterization of the antibodies is shown in supplementary Figure 1 in supplementary material available online at doi:10.1155/2009/267107.

#### 2.5.2. Reagents

Serum-free medium (SFM) plus was prepared by mixing DMEM (HG or NG) with 20 mM HEPES, 0.2% BSA, and 0.02% Sodium Azide. This was prepared immediately before use and kept on ice for the duration of the assay. A diethanolamine (DEA) buffer stock solution was prepared, stored at 4°C, and protected from light. For 500 mL of buffer, 4.8 mL of 85% diethanolamine (Fisher Cat no. D45) and 0.25 mL of 1 M MgCl_2_ were mixed with 995 mL of water. Immediately before use, the alkaline phosphatase substrate solution was prepared by mixing 20 mL of DEA buffer with one tablet of Para-nitrophenyl phosphate (pNPP) (Sigma Cat no. N2765).

#### 2.5.3. Treating Cells with Test Compounds

Prior to initiating the assays, treatments were prepared in SFM. For each assay 9 wells were incubated in NG-SFM alone and the remaining wells were incubated with HG-SFM glucose plus or minus test or control. The control compounds were (a) the heparin binding domain of vitronectin and (b) the IAP binding domain of thrombospondin. Both control compounds have been shown to inhibit IAP cleavage (data not shown). Cell monolayers were washed three times with serum free medium (SFM) and then 100 *μ*L of SFM plus or minus the treatments or controls was added. For each assay nine wells were rinsed and then SFM containing 5 mM glucose was added. Nine additional wells received HG-SFM alone and the remaining wells received either test or control compounds in HG-SFM. The 96 well plate was then placed in a 37°C incubator (5% CO_2_ and 80% humidity) for six hours. Each compound was tested at three concentrations (2, 5 and 10 *μ*M).

#### 2.5.4. Incubation with Primary Antibodies

After six hours the plate was placed on ice. SFM was aspirated from each well and the wells were rinsed three times with 100 *μ*L of SFM containing 0.2% bovine serum albumin, 20 mM HEPES and 0.0% sodium azide (SFM plus). Following rinsing with SFM plus the cell monolayer was incubated with the appropriate primary antibody prepared in SFM plus. For the nine control wells (incubated with either high- or normal-glucose media), three wells were exposed to 100 *μ*L of B6H12 (at a dilution of 1 : 100), the negative control antibody R250 (at a dilution of 1 : 100) or with the test antibody R593 (at a dilution of 1 : 100). The remaining wells were exposed to a 1 : 100 dilution of R593. The plates were then incubated overnight at 4°C with gentle rocking.

#### 2.5.5. Incubation with Secondary Antibodies

Following the overnight incubation the plates were placed on a tray of ice and each is well aspirated. They were rinsed three times with SFM plus prior to incubation with appropriate secondary antibody. Wells that had been exposed to B6H12 were incubated with 100 *μ*L of a 1 : 500 dilution of a goat antimouse alkaline phosphatase conjugated secondary antibody and the wells incubated with the R593 or R250 polyclonal antibodies were incubated with 100 *μ*L of a 1 : 500 dilution of the goat anti-Rabbit conjugated secondary antibody. The incubation proceeded for one hour at room temperature with gentle rocking.

#### 2.5.6. Addition of Alkaline Phosphate Substrate and Color Development

5 minutes before the end of the one hour incubation the alkaline phosphatase substrate developing solution was prepared. After one hour the secondary antibody was removed and the wells were washed three times with SFM plus. 50 *μ*L of the alkaline phosphatase developing solution was then added to each well and the color was allowed to develop being protected from light for 20 minutes at room temperature. The yellow color of nitrophenol was measured at 405 nm giving an optical density (OD) reading for each treatment.

#### 2.5.7. Data Analysis

The OD reading determined after antibody binding to SMC exposed to HG was subtracted from the OD reading for antibody binding to SMC exposed to NG. This value was referred to as ΔOD^max^. The OD reading for antibody binding to SMC treated with HG was then subtracted from the OD reading for each compound (ΔOD^test^). A percent score for each compound was then calculated by the following equation: ΔOD^test ^/ΔOD^max^ × 100.

#### 2.5.8. Criteria for Identification of Positive Hit

A compound was considered active if, in the presence of the compound, the increase in the amount of antibody (that specifically recognizes cleaved IAP) bound to cells in HG was at least 75% of the value obtained when antibody binding was quantified in NG. Therefore the percent score is a measure of the ability of the compound (at 10 *μ*M) to increase antibody binding. The compounds that achieved this result were then tested over a range from 0.2 *μ*M to 10 *μ*M (0.2, 0.5, 2, 5, 10 *μ*M*).* The concentration of compound in which the increase in antibody binding to cells in high glucose was 50% of the difference between antibody binding to cells in normal glucose as compared to high glucose was considered the ED_50_.

### 2.6. Immunoblotting to Validate Primary Screening Results

To confirm that the compounds that increased antibody binding in the ELISA could alter IAP cleavage, REC or SMC that had been maintained in HG-GM were exposed to the compounds then lysates were prepared and analyzed by immunoblotting using either the anti-IAP antibody that only recognizes intact IAP (R569) or the monoclonal antibody B6H12 that recognizes both intact and the residual membrane associated fragment of IAP ([31] and supplementary Figure 1). REC and SMC were plated in 10 cm dishes in HG- or NG-GM and grown to confluency for 7 days with the medium being changed every 2-3 days. On day 7 the growth medium was removed and the confluent monolayers were rinsed three times with SFM-HG or NG then incubated overnight (16-17 hours). The compounds were then added for 6 hours. Cells were lysed in a modified RIPA buffer. Following centrifugation, equal amounts of cellular protein (as determined using the BCA protein assay (Pierce) data not shown) were mixed with nonreducing gel loading buffer, heated to 70°C for 10 minutes and separated by SDS-PAGE (8%). Following SDS-PAGE, IAP was visualized by immunoblotting as we have previously described [33].

### 2.7. Assays to Determine Whether Compounds That Accelerate IAP Cleavage Inhibit IGF-I Signaling in High Glucose

#### 2.7.1. Cell Proliferation

Cell proliferation assays were performed as we have described previously [36]. SMCs were plated at 2 × 10^4^ cells per well in each well of a 24 well plate in HG-SFM or NG-SFM plus 2% FBS. Cells were allowed to attach overnight before the medium was replaced with fresh SFM. 24 hours later the SFM was replaced with SFM + 0.2% platelet poor plasma (PPP) with or without the addition of IGF-I (50 ng/mL). The compounds were added at a concentration of 5 *μ*M immediately prior to the addition of IGF-I. Cell number was determined following trypsinization, trypan blue staining and counting [29].

#### 2.7.2. Cell Lysis, Immunoprecipitation and Western Immunoblotting

REC and SMC cultures were exposed to test reagents as described above for the secondary screening assay. Immunoprecipitation studies (using anti-SHPS-1 and Shc antibodies) were performed as previously described [33]. Following SDS-PAGE the proteins were visualized by immunoblotting as we have previously described [33]. For immunoblotting the antibodies (B6H12, R569, SHPS-1, p-Tyr, Shc, pERK1/2) were used at concentrations between 1 : 500 and 1 : 1000.

### 2.8. Statistical Analysis

Chemiluminescent images obtained were scanned using a DuoScan T1200 (AGFA Brussels, Belgium) and band intensities of the scanned images were analyzed using NIH Image, version 1.61. The Student's *t*-test was used to compare differences between treatments. The results that are shown, expressed as the mean ± SEM, are representative of at least three separate experiments.

## 3. Results

### 3.1. Primary Screening

The ELISA that measures the degree of proteolytic cleavage of IAP in the presence and absence of test compounds in SMC in 25 mM glucose relies on an antibody (R593) that recognizes a neo-epitope whose affinity for the antibody is increased following cleavage (supplementary Figure 1). Of the 1040 compounds tested 14 were considered “positive hits” according to the criteria described in Section 2. These are listed in Table 1. Further analysis of these compounds allowed determination of the ED_50_ and the effective concentration range (Table 1).

### 3.2. Secondary Screening (SMC)

**Figure 1:**
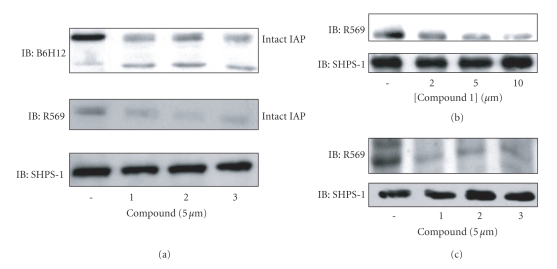
*Acceleration of IAP cleavage by positive hit compounds (a) and (b)*. SMCs were grown to confluency in DMEM containing 25 mM glucose and then incubated overnight in SFM. They were then incubated in HG-SFM alone or HG-SFM containing the test compounds (2, 5 or 10 *μ*M) for 6 hours. Following lysis and separation by SDS-PAGE the amount of intact IAP was detected by western immunoblotting with the anti-IAP monoclonal antibody, B6H12 which recognizes intact and residual membrane associated IAP fragment or the anti-IAP antibody R569 which specifically recognizes intact IAP. To control for differences in protein equal amounts of lysate were also immunoblotted with the anti-SHPS-1 antibody. (c) REC were maintained in medium containing 25 mM glucose prior to incubation overnight in SFM. The test compounds were added at a concentration of 5 *μ*M for 6 hours. IAP was visualized as described above.

In contrast to the primary screening assay that utilized an antibody that specifically detected IAP cleavage, the secondary screening assays used one of two antibodies, R569, that binds to the cleaved portion of IAP and thus does not recognize the residual transmembrane fragment that remains following cleavage or the anti-IAP monoclonal antibody, B6H12, which recognizes both intact and the residual membrane fragment [31]. The results from these assays were determined by western immunoblotting rather than ELISA. Treatment of SMC with each of the 14 compounds resulted in significant decrease in the amount of intact IAP that could be detected (Figure 1(a) shows an example of the results obtained using compounds 1–3 and the data are summarized for all 14 compounds in Table 2).

### 3.3. Secondary Screening (REC)

We then tested compounds 1 through 3 for their ability to accelerate IAP cleavage in REC grown in high-glucose medium (Figure 1(b)). All 3 compounds significantly decreased the amount of intact IAP that could be detected compared with REC grown in high glucose.

### 3.4. Effect of “Positive Hit” Compounds on IAP Association with SHPS-1

Our previous studies have shown that in order for IGF-I to stimulate an increase in both REC and SMC migration and proliferation a specific signaling complex must be formed. Specifically, the two transmembrane proteins, IAP and SHPS-1 must associate, via their extracellular domains which permits SHPS-1 phosphorylation in response to IGF-I [27]. Phosphorylation of SHPS-1 is necessary for the formation of the SHP-2-Src-Shc complex which leads to the subsequent phosphorylation of Shc and activation of the MAP and PI-3 kinase signaling pathways since both of which are required for IGF-I-stimulated migration and proliferation [29]. When RECs or SMCs are maintained in 5 mM glucose SHPS-1 is not phosphorylated therefore this complex does not form due to the cleavage of IAP [30, 31]. Thus, to determine the effect of the compounds that accelerate IAP cleavage on IGF-I signaling we examined formation of the IAP-SHPS-1 signaling complex.

**Figure 2:**
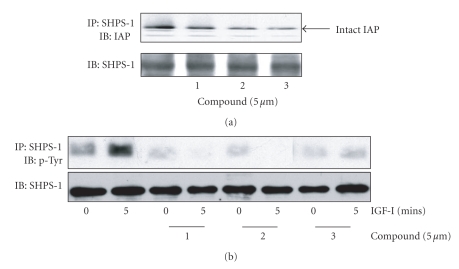
*Acceleration of IAP cleavage inhibits IAP-SHPS-1 association and SHPS-1 phosphorylation*. SMCs were grown to confluency in DMEM containing 25 mM glucose and then incubated overnight in SFM. They were then incubated in HG-SFM or HG-SFM containing the test compounds (mM) for 6 hours. Where indicated the cultures were treated with IGF-I (100 ng/mL) for 5 minutes prior to lysis. Cell lysates were then immunoprecipitated and proteins visualized by western immunoblotting. To control for differences in total protein samples were also immunoblotted with an anti-SHPS-1 antibody. (a) IAP association with SHPS-1 was determined following immunoprecipitation (IP) with an anti-SHPS-1 antibody and immunoblotting (IB) with the anti-IAP monoclonal antibody, B6H12. (b) SHPS-1 phosphorylation in response to IGF-I was determined by IP with an anti-SHPS-1 antibody and IB with an antiphosphotyrosine antibody (p-Tyr). To control for differences in total protein samples were also immunoblotted with an anti-SHPS-1 antibody.

Each compound significantly reduced the association of IAP with SHPS-1 (Figure 2(a) and Table 2). The significance of IAP-SHPS-1 disruption was assessed by measuring SHPS-1 phosphorylation. In the presence of each compound the ability of IGF-I to stimulate SHPS-1 phosphorylation was significantly impaired (Figure 2(b) and Table 2).

**Figure 3:**
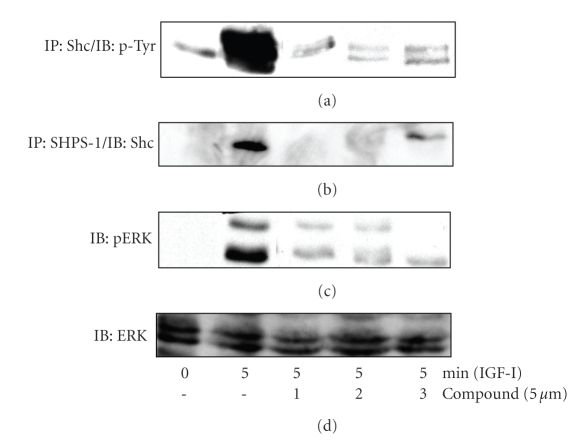
*Acceleration of IAP cleavage inhibits signaling in response to IGF-I*. SMCs were grown to confluency in DMEM containing 25 mM glucose and then incubated overnight in HG-SFM. SMC were then incubated in HG-SFM, NG-SFM (5 mM) or HG-SFM containing the test compounds (*μ*M) for 6 hours. Where indicated the cultures were treated with IGF-I (100 ng/mL) for 5 minutes prior to lysis. Cell lysates were then used for immunoprecipitation and proteins visualized by western immunoblotting. (a) Shc phosphorylation in response to IGF-I was determined by IP with an anti-Shc antibody and IB with an antiphosphotyrosine antibody (p-Tyr). (b) Shc association with SHPS-1 was determined following immunoprecipitation (IP) with an anti-SHPS-1 antibody and immunoblotting (IB) with an anti-Shc antibody. (c) MAPK activation was assessed by immunoblotting lysates directly with an antibody that specifically recognizes the phosphorylated forms of ERK1/2 (pERK). (d) To control for loading samples were immunoblotted with an anti-ERK antibody.

The disruption in IAP-SHPS-1 association and inhibition of SHPS-1 phosphorylation would be predicted to inhibit downstream signaling. Each compound significantly inhibited IGF-I stimulation of Shc phosphorylation (Figure 3(a) and Table 2) and Shc recruitment to SHPS-1 (Figure 3(b) and Table 2). Our previous studies have shown that the phosphorylation of Shc is required for activation of downstream signaling pathways including the MAPK pathway. Consistent with the significant decrease in Shc phosphorylation, activation of MAPK, as shown by analysis of the phosphorylated forms of ERK1/2 in response to IGF-I, was also significantly impaired in the presence of each of the 14 compounds (Figure 3(c) and Table 2).

**Figure 4:**
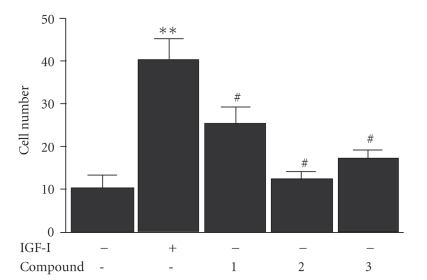
*Inhibition of IGF-I-stimulated SMC proliferation*. 2 × 10^4^ cells were plated in each well of a 24 well plate prior to exposure to IGF-I (50 ng/mL), and the test compounds (all prepared in DMEM-H + 0.2% platelet poor plasma). 48 hours after the addition of IGF-I (50ng/mL) cell number was determined by trypan blue staining and counting. *P < .05 when cell in response to IGF-I is compared with the number of cells in DMEM-H alone.

In 25 mM glucose these changes led to enhanced SMC proliferation in response to IGF-I. When SMCs were maintained in 25 mM glucose IGF-I-stimulated a significant, 3 fold increase in SMC proliferation. In contrast, the addition of each compound resulted in at least a 2 fold decrease in IGF-I-stimulated proliferation (Figure 4 and Table 2).

## 4. Discussion

Atherosclerosis represents the leading cause of death in both type 1 and type 2 diabetes [37–39]. Intensive multifactorial treatment of hyperglycemia, dyslipidemia and hypertension can significantly reduce the incidence of cardiovascular disease but only a small percentage of patients is able to manage all these factors to achieve sufficient control to lower their risk [40]. Thus there is a need for a systemic therapy that inhibits the progression of the disease in these patients. Proliferative retinopathy is one of the most severe and prevalent complications of diabetes. 34 years after disease onset patients who were diagnosed prior to age 30 have a prevalence of 70%. Even with improved glycemic control the probability of developing diabetic retinopathy after having had type 1diabetes for 30 years is greater than 60%. Because of this high prevalence diabetic retinopathy remains the second leading cause of blindness in individuals over 60 years of age. As patients with type 1 diabetes continue to live longer the prevalence of loss of vision due to diabetic retinopathy is likely to increase. Therefore there will continue to be a need in patients with type 1 diabetes for this type of therapeutic approach. Treatment of this disorder has been through either technical procedures such as photocoagulation [41] and vitrectomy [42] or preventative measures such as reduction in hyperglycemia [43] and blood pressure [44]. Three therapies have been introduced based on inhibiting pathophysiologic changes in biochemical signaling pathways. The most effective is inhibition of vascular endothelial growth factor (VEGF), a mitogen for retinal vein endothelial cells[45]. When VEGF antibodies are directly injected into eyes of human subjects they limit the proliferative phase of the disease [46]. Similarly enhanced activation of the protein kinase C beta pathway is present and an orally active, PKC beta inhibitor reduces retinopathy progression [47]. Treatments which lower serum IGF-I concentrations have been tried but because of their relative ineffectiveness in lowering serum IGF-I concentrations they had limited efficacy [48].

The focus of our studies has been to identify prevention and treatment strategies for the vascular complications that are associated with diabetes by identifying signaling events that are specific for the hyperglycemic environment. Identification of such targets has the potential for the development of therapies that will prevent the disease associated events without disrupting the normal function of the cells.

Increased activity of IGF-I has been implicated in both atherosclerosis and diabetic retinopathy. Since our previous studies have shown that both REC and SMC grown in high glucose are more responsive than those grown in normal glucose [26] and that the difference in responsiveness is due to regulation of IAP cleavage [30, 31] we hypothesized that acceleration of IAP cleavage was a potential strategy for the inhibition of IGF-I-stimulated vascular cell proliferation specifically in hyperglycemic conditions. In this study we screened 1040 compounds for their ability to accelerate IAP cleavage in a cell-based ELISA using an antibody specific for the cleaved form of IAP. While the extent of IAP cleavage stimulated by the addition of each compound varied, in each case the extent of IAP cleavage was sufficient to significantly disrupt IAP-SHPS-1 association. We have shown previously that disrupting IAP-SHPS-1 association with the anti-IAP antibody, B6H12, inhibits IGF-I-stimulated signaling [27]. The results show that each compound inhibited SHPS-1 phosphorylation [27]. Additionally, the recruitment and phosphorylation of Shc which is necessary for activation of downstream signaling pathways [29] in response to IGF-I was significantly impaired in the presence of each of the active compounds. Given the importance of IAP in regulating the response of REC and SMC to IGF-I it is reasonable to conclude that identification of compounds that can disrupt the hyperglycemia induced increase in IAP-SHPS-1 association has the potential to inhibit IGF-I signaling in vascular cells in vivo.

Of the most active compounds they could be divided into three groups. Both Pyridostigmine bromide and Cephaloridine are quarternary pyridinium compounds. The second group are all dipeptide analogs (e.g., n-formyl methionyl leucly phenylalanine). The third group are arylalkylamino compounds including chloropromazine, dopamine, hydralazine and mephenentermine. Studies will now be required to identify the molecular pathways stimulated by each compound that mediate their effects on IAP cleavage. In particular, it will be of interest to determine whether there is convergence between signaling pathways triggered by the diverse group of compounds identified. 

We have determined that the protease responsible for the cleavage of IAP when SMCs are maintained in normal glucose is MMP-2 [31]. Conversion of MMP-2 to the fully-active form is a multistep process involving the formation of a complex including the membrane-bound MT1-MMP which binds to TIMP-2 which in turn binds to pro-MMP-2. This complex then catalyzes the conversion of MMP-2 to the intermediate and then active form of the enzyme [49–56]. Further studies will be required to determine whether these compounds alter the amount of MMP-2 and TIMP-2.

While our data support our conclusion that the inhibitory effect of each compound is mediated by its effects on IAP cleavage, we can not rule out the possibility that the compounds also have additional inhibitory effects on the IGF-I signaling pathways that are not mediated through acceleration of IAP cleavage. IAP is a binding site for the matricellular protein Thrombospondin-1 (TS-1) [33]. We have shown previously that there is a significant increase in TS-1 deposition in the matrix surrounding SMC cultured in high glucose compared with that of normal glucose and that TS-1 is a potent stimulator of IGF-I signaling [26, 33]. One alternative mechanism distinct from the regulation of IAP cleavage by MMP-2 is the possibility that the compounds regulate TS-1 association with IAP and thereby regulating its susceptibility to cleavage.

The results of these studies indicate that in vivo studies are now warranted to determine the efficacy of the positive hit compounds at inhibiting IGF-I-stimulated REC and/or SMC proliferation in vivo. We have shown that inhibition of IAP association with SHPS-1 in diabetic mice results in attenuation of SMC proliferation [32]. Since acceleration of IAP cleavage led to a similar change in IGF-I activity in vitro our findings support the conclusion that these compounds will be effective in vivo in inhibiting SMC responses to IGF-I. A therapy that accelerates IAP cleavage would be designed to reverse a qualitatively distinct pathophysiologic effect of hyperglycemia and thus should allow normal maintenance of IGF-I signaling in vascular cells.

In conclusion, in this study we developed a cell-based ELISA to allow the high-throughput screening of small molecules to identify those that accelerated IAP cleavage. This assay is now available for the screening of second generation compounds derived from the most promising lead compounds. Given the in vitro data obtained with the active compounds it seems highly probable that with further development and testing we could conclude a novel compound could be identified when used either alone that or in combination with other therapies to prevent or treat vascular complications associated with diabetes.

## Supplementary Material

Supplemental Figure 1: To determine whether cleavage resulted in loss of the extracellular domain the anti IAP polyclonal antibody (R569) raised using a peptide that contained amino acids 43–61 of IAP as an immunogen was used (top panel). To detect the fragment shed after cleavage, conditioned media samples were concentrated and then IAP was visualized using the anti IAP antibody R569 (second panel). The residual cell associated fragment of IAP was detected following immunoblotting with the anti IAP polyclonal antibody (R593). This antibody was raised using a peptide containing amino acids 75–94 of IAP (third panel). The membranes were stripped and reprobed with an anti SHPS-1 antibody (bottom panel). The graph shows the difference in intact IAP detected with R569 in SMCs grown in 25 mM glucose and those grown in 5 mM glucose expressed as arbitrary scanning units (mean ± sem, *n* = 3, * *P* < .05).

## Figures and Tables

**Table 1:** Ranking of compounds that accelerate IAP cleavage.

Number	Compound	ED_50_ (*μ*M)	Active range (*μ*M)	Mean % increase in R593 binding (5 *μ*M)
1	l-leucyl alanine	0.5	0.2–10	96 ± 0.8
2	n-formyl methionyl leucyl phenylalanine	0.75	0.2–10	100 ± 5
3	Mephentermine sulfate	1	0.2–10	90 ± 5
4	Lysyl tyrosyl lysine acetate	2	0.2–10	75 ± 20
5	Dopamine hydrochloride	2	1–10	84 ± 8
6	N formyl methionyl phenylalanine	2	1–10	75 ± 5
7	Dyclonine hydrochloride	2.5	1–10	92 ± 8
8	Amoxicillin	3	1–10	85 ± 2
9	Hydralazine hydrochloride	3	1–10	76 ± 7.5
10	Cephaloride	4	2–10	65 ± 5
11	Dinitolmide	5	2–10	35 ± 12
12	Acetyl cysteine	6	2–10	30 ± 5
13	Pyridostigmine bromide	6.5	1–10	30 ± 1
14	Chloropromazine	7	2–10	25 ± 2

**Table 2:** Effect of compounds on IAP cleavage, IAP-SHPS-1 association and IGF-I-stimulated signaling events.

Compound number	Decrease in R569 binding*	IAP-SHPS-1 association**	SHPS-1 phosphor**	SHPS-1-Shc association**	Shc phosphor**	ERK Phosphor**
1	97 ± 2	80 ± 5	87.5 ± 12	96.5 ± 3	76 ± 8	93 ± 5
2	91 ± 1.5	97 ± 2	93 ± 7	95.5 ± 4	77 ± 13	84 ± 14
3	85 ± 3	100 ± 0	99 ± 1	89 ± 0.5	81 ± 10	84 ± 9
4	63 ± 18	58 ± 15	94 ± 5	85 ± 5	65 ± 10	79 ± 13
5	80 ± 6	52 ± 1	77 ± 14	90 ± 2	83 ± 4	70 ± 13
6	78 ± 6	55 ± 10	90 ± 5	93 ± 1.5	85 ± 7	65 ± 15
7	83 ± 3	92 ± 1	76 ± 14	94 ± 3	80 ± 20	85 ± 12
8	78 ± 1	55 ± 5	76 ± 7	84 ± 7	66 ± 16	92 ± 1
9	70 ± 8	21 ± 2	50 ± 28	84 ± 6	90 ± 5	73 ± 12
10	78 ± 2.5	90 ± 8	85 ± 8	86 ± 5	57 ± 7	82 ± 8
11	50 ± 5	60 ± 12	90 ± 4	75 ± 10	65 ± 15	90 ± 4
12	78 ± 4.5	55 ± 5	89 ± 11	78 ± 2	78 ± 2	99 ± 1
13	45 ± 5	97 ± 1	90 ± 10	84 ± 3	73 ± 4	89 ± 4
14	72 ± 4	66 ± 7	47 ± 5	67 ± 2.5	77 ± 3	92 ± 1

*% Decrease in R569 antibody binding compared with SMC maintained in 25 mM glucose.

**% Decrease in association, phosphorylation or recruitment in response to IGF-I compared with SMC maintained in 25 mM glucose.

Results shown are mean ± sem *N* = 3.
