# Supplementary material for: Identification of Compounds That Inhibit IGF-I Signaling in Hyperglycemia
Source: Exp Diabetes Res. 2010 Jan 6;2009:267107. doi: 10.1155/2009/267107 (PMC2810469; doi:10.1155/2009/267107)
Supplement: Supplementary file 1 — Supplemental Figure 1: To determine whether cleavage resulted in loss of the extracellular domain the anti IAP polyclonal antibody (R569) raised using a peptide that contained amino acids 43–61 of IAP as an immunogen was used (top panel). To detect the fragment shed after cleavage, conditioned media samples were concentrated and then IAP was visualized using the anti IAP antibody R569 (second panel). The residual cell associated fragment of IAP was detected following immunoblotting with the anti IAP polyclonal antibody (R593). This antibody was raised using a peptide containing amino acids 75–94 of IAP (third panel). The membranes were stripped and reprobed with an anti SHPS-1 antibody (bottom panel). The graph shows the difference in intact IAP detected with R569 in SMCs grown in 25 mM glucose and those grown in 5 mM glucose expressed as arbitrary scanning units (mean ± sem, n = 3, * P < .05). [file 267107.f1.pdf]

Supplemental Figure 1: To determine whether cleavage resulted in loss of the extracellular domain the anti IAP polyclonal antibody (R569) raised using a peptide that contained amino acids 43 - 61 of IAP as an immunogen was used (top panel). To detect the fragment shed after cleavage, conditioned media samples were concentrated and then IAP was visualized using the anti IAP antibody R569 (second panel). The residual cell associated fragment of IAP was detected following immunoblotting with the anti IAP polyclonal antibody (R593). This antibody was raised using a peptide containing amino acids 75 – 94 of IAP (third panel). The membranes were stripped and reprobed with an anti SHPS-1 antibody (bottom panel).

The graph shows the difference in intact IAP detected with R569 in SMCs grown in 25 mM glucose and those grown in 5 mM glucose expressed as arbitrary scanning units (mean ± sem, n =3, * $p < 0.05$).

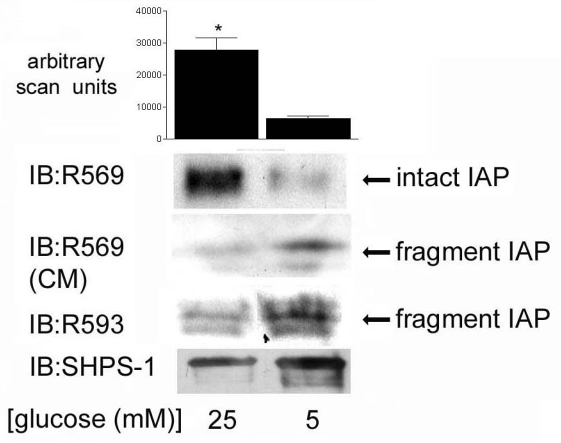

Supplemental Figure 1
